# High Adherence to the Nordic Diet Is Associated with Lower Levels of Total and Platelet-Derived Circulating Microvesicles in a Norwegian Population

**DOI:** 10.3390/nu11051114

**Published:** 2019-05-18

**Authors:** Gemma Chiva-Blanch, Kristian Laake, Peder Myhre, Vibeke Bratseth, Harald Arnesen, Svein Solheim, Lina Badimon, Ingebjørg Seljeflot

**Affiliations:** 1 Center for Clinical Heart Research, Department of Cardiology, Oslo University Hospital Ullevål, 0450 Oslo, Norway; kristian.laake@medisin.uio.no (K.L.); pedermyhre@gmail.com (P.M.); vbratset@ous-hf.no (V.B.); UXHAAR@ous-hf.no (H.A.); svein.solheim@ulleval.no (S.S.); UXINLJ@ous-hf.no (I.S.); 2 Cardiovascular Program-ICCC, Research Institute- Hospital Santa Creu i Sant Pau, 08025 Barcelona, Spain; LBadimon@santpau.cat; 3 Faculty of Medicine, University of Oslo, 0372 Oslo, Norway

**Keywords:** Nordic diet, circulating microvesicles, SmartDiet, cardiovascular disease, platelets

## Abstract

Circulating microvesicles (cMV) are small phospholipid-rich blebs shed from the membrane of activated vascular cells that contribute to vascular disease progression. We aimed to investigate whether the quality of the Nordic diet is associated with the degree of blood and vascular cell activation measured by MV shedding in elderly patients after an acute myocardial infarction (AMI). One-hundred and seventy-four patients aged 70–82 years were included in this cross-sectional study. Fasting blood samples were taken within 2 to 8 weeks after an AMI. Annexin V (AV)^+^ cMV derived from blood and vascular cells were measured through flow cytometry. A patient’s usual diet was recorded with the SmartDiet® questionnaire. Patients with higher adherence to the Nordic diet (highest diet score) had lower levels of total AV^+^ and platelet-derived (CD61^+^/AV^+^ and CD31^+^/AV^+^) cMV. Dietary habits influence cellular activation. A high adherence to the Nordic diet (assessed by the SmartDiet® score) in elderly post-AMI patients was associated with lower levels of platelet activation, which was reflected by a lesser release of MV carrying platelet-derived epitopes, potentially contributing to an explanation of the cardioprotective effects of the Nordic diet.

## 1. Introduction

Cardiovascular disease (CVD) is one of the main causes of death worldwide. The relationship between diets and CVD is complex, although it has been shown that some dietary patterns, such as the Mediterranean diet [1] or the Nordic diet [2], elicit cardioprotective effects.

The Nordic diet is characterized by a high intake of foods produced locally, such as salmon and herring (fatty fish); cod; tubercles; pulses; wholegrain cereals such as barley, oats, and rye; berries (which are very rich in polyphenols); and other fruits. Therefore, this diet is especially rich in long-chain omega 3 fatty acids, accompanied by a moderate-to-high intake of fiber, minerals, and antioxidants, and has been shown to improve endothelial function and alleviate proinflammatory states [2]. Over recent years, this dietary pattern has been the object of increasing interest because it has been associated with decreased overall and cancer mortality, although controversial results have been observed in terms of CVD mortality [3].

Circulating microvesicles (cMV) are a subtype of extracellular vesicles rich in phospholipids that range from 0.1 to 1 micrometer (µm) diameter and shed from cells when activated, injured, or undergoing apoptotic or necrotic processes. Circulating microvesicles act as procoagulant agents because an externalization of the procoagulant anionic phosphatidylserine (PS) takes place during MV formation. In addition, cMV carry antigens from their cell of origin and have also been found to be involved in inflammatory processes, thus contributing to both the initiation and progression of CVD. In fact, elevated platelet-, endothelial-, and leukocyte-derived cMV have been observed in several CVD states and have been associated with several CVD risk factors, as reviewed in Reference [4]. The effects of diet on MV shedding have been scarcely investigated (reviewed in Reference [5]), and the relationship between the Nordic diet and MV generation is largely unknown. Therefore, the aim of our study was to investigate whether the quality of the Nordic diet is associated with MV shedding in elderly patients after an acute myocardial infarction (AMI), which is known to increase the levels of cMV [6,7].

## 2. Materials and Methods

### 2.1. Study Population

The subjects included in this cross-sectional study belong to a subset of the “OMega-3 fatty acids in Elderly patients with Myocardial Infarction” (OMEMI; NCT01841944, Clinical-Trials.gov) cohort [8] at inclusion in the study. The protocol was approved by the Regional Committee for Medical Research Ethics (ref. number: 2012/1422). Patients 70–82 years old were recruited at Oslo University Hospital Ullevål, Oslo, Norway, after a diagnosis of an AMI, and were included in the study after 2 to 8 weeks of surviving the AMI. Patients were treated per guidelines with standard medication. Demographic data, clinical characteristics, cardiovascular history, classic cardiovascular risk factors, and current pharmacological treatment were obtained from all subjects using a standardized report form at inclusion. A patient’s usual diet was recorded at study entry with the SmartDiet® questionnaire (version 3, from May 2009), which is a fast, simple, and validated tool for the assessment of food intake and diet quality, and results in a diet score [9]. As shown in Table 1, the SmartDiet score is built upon 14 questions relating to food quantity and quality, which are scored, summed, and categorized as unhealthy (≤ 27 points, group I), somewhat unhealthy (28–35 points, group II), or healthy diet (≥ 36 points, group III), according to the Nordic Nutrition Recommendations. The study protocol was approved by the Regional Committee for Medical Research Ethics and followed the principles outlined in the Declaration of Helsinki. All participants signed their informed consent.

### 2.2. Blood Sampling

Fasting venous blood was withdrawn into 3.8% sodium citrate tubes for MV analysis. Blood cells were removed by centrifuging whole blood 20 min at 2500× *g* at 4 ºC. Plasma was carefully recollected, leaving about 0.2 cm of an undisturbed layer on top of the cells, and aliquots were immediately frozen and stored at −80 ºC until processing for isolation and quantification of cMV. Additionally, biochemical and hematological parameters were quantified with standardized routine methods.

### 2.3. Circulating Microvesicle Isolation and Quantification

The cMV fraction was isolated from citrated and anticoagulated plasma through a two-step high-speed centrifugation procedure. Five-hundred microliters (µL) of frozen plasma aliquots were thawed on melting ice and centrifuged again at 2500× *g* for 15 min at room temperature (RT) [10]. Then, 250 µL of plasma were collected and centrifuged at 20000× *g* for 30 min at RT to pellet cMV. The supernatants were discarded, and the cMV-enriched pellet was washed once with citrate-phosphate buffered saline (PBS) solution (citrate-PBS; 1.4 mM phosphate, 154 mM NaCl, 10.9 mM trisodium citrate, pH 7.4) before a second equal centrifugation step was made. Finally, the remaining cMV pellets were resuspended in 100 µL of citrate-PBS.

A triple-label flow cytometry analysis was performed as described before [7,11]. Briefly, 5 µL of washed cMV suspensions were diluted in 30 µL of PBS buffer containing 2.5 mM of CaCl_2_ (Annexin Binding Buffer, ABB). Thereafter, combinations of 5 µL of allophycocyanin (APC)-conjugated Annexin V (AV, BD-horizon) with two specific monoclonal antibodies (mAb, 5 µL each, Table 2) labeled with fluorescein isothiocyanate (FITC) and phycoerythrin (PE) (or the isotype-matched control antibodies) were added.

Samples were incubated for 20 min at RT in the dark and diluted with ABB before being immediately analyzed with the “Auto Collect” mode in 96-well plates on an AccuriC6 flow cytometer (BD, Accuri® Cytometers, Inc., San Diego, CA, US), except for MV from smooth muscle cells (SMCs), which were quantified as previously described [6,7]. Specifically, for the detection and quantification of cMV from SMC origin, 5 µL of the cMV suspension were incubated for 20 min at RT in the dark with 5 µL of AV-APC and 5 µL of CD142-FITC (tissue factor, TF) in a final volume of 50 µL ABB. cMV were fixed with 250 µL of ABB/paraformaldehyde 2% for 30 min and centrifuged at 20000× *g* for 30 min to pellet cMV. After eliminating the supernatant, cMV were permeabilized with 45 µL of ABB/saponin 0.1% for 20 min at RT in the dark. After permeabilizing, 5 µL of smooth muscle actin (SMA)-α-PE were added to the cMV suspension, incubated for 20 min at RT in the dark, and finally diluted with ABB prior to flow cytometer analyses.

**Figure 1:**
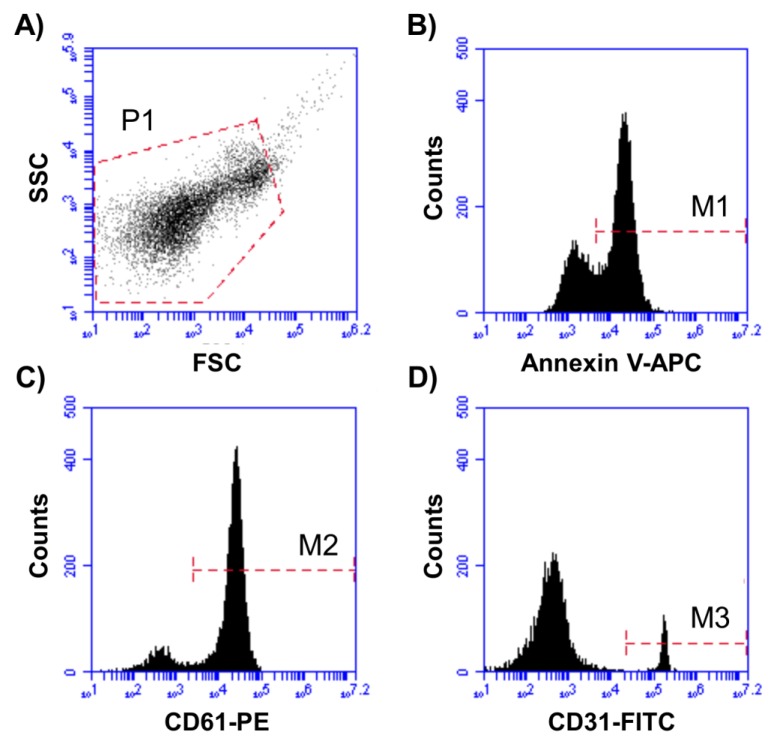
Representative plots for MV identification and characterization. (**A**) P1 was set according to cMV size and granularity (defined as <1 µm). (**B**) Annexin V-APC^+^ cMV (M1) were selected from P1. (**C**) AV^+^ cMV binding PE^+^ (M2)- or (**D**) FITC^+^ (M3)-labeled antibodies were selected from P1 and quantified. Double staining with FITC- and PE-labeled antibodies from M1 (Annexin V^+^ cMV) was also quantified. Here, cMV indicates circulating microvesicles, APC denotes allophycocyanin, FITC indicates fluorescein isothiocyanate, and PE is phycoerythrin.

For all cMV characterization and quantification, acquisition was performed at 2 min per sample. The flow rate was set at 14 µL/min. Forward scatter (FSC), side scatter (SSC), and fluorescence data were obtained with the settings on the logarithmic scale. In addition, cMV were identified and quantified based on their binding to AV and their reactivity to cell-specific mAbs (Figure 1). To identify positive marked events, thresholds of fluorescence were set based on samples incubated with the same final concentration of isotype-matched control antibodies after titration experiments. The AV binding level was corrected for autofluorescence using fluorescence signals obtained with MV labeled with AV in a calcium-free buffer (PBS). To reduce background noise, buffers were prepared on the same day and filtered through 0.2-µm pore size filters under vacuum.

Data were analyzed with BD CSampler software (version 1.0.264.21, Accuri® Cytometers, Inc.). The cMV concentration (number of cMV per µL of plasma) was determined according to Nieuwland’s formula [12].

### 2.4. Statistical Analysis

Statistical analyses were performed using the SPSS Statistical Analysis System (version 23.0, IBM Corp. Armonk, NY). Descriptive statistics (mean ± SD or *n* (%)) were used to describe the characteristics of the patients and the outcome variables. The normality of the variables was assessed with the Shapiro–Wilk test. All variables with a skewed distribution were transformed into their natural logarithms for regression analyses. ANOVA with the Bonferroni post hoc test or *t*-test analyses were conducted to examine the differences in MV concentrations related to cardiovascular risk factors and potential confounding factors and related to groups of the SmartDiet score. A linear regression was performed to investigate the specific influence of covariates in the relationship between diet and cMV. According to the results, we performed Spearman’s correlations and multivariate linear regressions adjusted for age, sex, use of fish oil supplements, and smoking history to analyze the main associations between the SmartDiet score and cMV. A two-tail *p*-value of <0.05 was considered statistically significant.

## 3. Results

### 3.1. Patients’ Characteristics 

Patients were divided into three categories according to the SmartDiet score, which was based on the usual intake of the different food groups included in the SmartDiet questionnaire. Group I was defined as ≤ 27 points (*n* = 39, 22.4%), group II as 28–35 points (*n* = 106, 60.9%), and group III as ≥ 36 points (*n* = 29, 16.7%) in the questionnaire, where the higher the score, the healthier the diet, according to the Nordic Nutrition Recommendations [9]. Table 3 shows the characteristics of the 174 patients included in the study according to groups of score. Although cMV concentrations from endothelial cells, erythrocytes, lymphocytes, and monocytes increased over time after an AMI (not shown), the distribution of patients according to the weeks after an AMI was homogeneous within tertiles of adherence to the Nordic diet. Therefore, no statistical interaction was observed between adherence to the Nordic diet and weeks after the AMI at inclusion in the study, for the levels of cMV in the patients studied. The mean age was 75 years, and approximately 60% were hypertensive. Almost all patients were under statin and dual antiplatelet therapy (98% and 95.5%, respectively), 24% were diabetics, 10% were current smokers, and 50% of the study population used fish oil supplements prior to inclusion.

**Figure 2:**
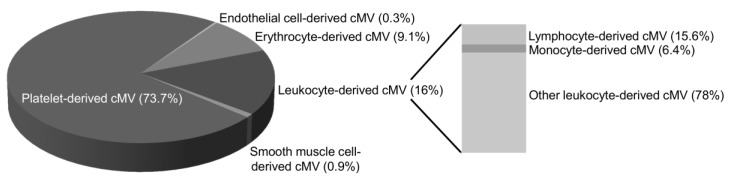
Distribution of cell origins of circulating microvesicles in the 174 patients studied. The pie charts show the distribution of cMV from the patients studied (*n* = 174) by major cell origins, indicated by percentages of each marker relative to cell lineage. Only cMV that were Annexin V-positive were considered. The used markers were CD61 for platelets, CD146 for endothelial cells, CD235ab for erythrocytes, CD45 for total leukocytes, CD3 for lymphocytes, CD14 for monocytes, and SMA-α for smooth muscle cell origins. Other leukocyte-derived cMV were positive for CD45 but negative for CD3 or CD14 staining. The markers used for MV characterization are defined in Table 2. Here, cMV indicates circulating microvesicles.

Figure 2 depicts the main cell origin of cMV. No significant differences were observed in the percentage of cMV from each cell origin or in clinical and biochemical characteristics of patients according to groups of diet score.

### 3.2. Food Items and SmartDiet Score

In general terms, the mean score for each item (Table 4) was higher in group III compared to group II and group I, and also was higher in group II compared to group I for nearly each food item, except for the “mayonnaise, remoulade, and kaviar” and “sweet toppings and sweet drinks” food items, in which no differences were observed between the three groups of the SmartDiet score. Hence, patients in group III had a higher score for almost every food item (12 out of 14) of the questionnaire compared to patients allocated to group II or group I.

### 3.3. Associations between Circulating Microvesicles and SmartDiet Score

The Spearman’s analyses showed an inverse correlation between the SmartDiet score and total AV^+^, and CD61^+^/AV^+^ and CD31^+^/AV^+^ cMV, reflecting platelet activation (*R* = −0.162, −0.176, and −0.159, and *p* = 0.045, 0.029, and 0.049, respectively). Thus, the association between SmartDiet score and these cMV was further analyzed by linear regression, adjusting for age, sex, use of fish oil supplements, and smoking history. Multivariable adjustments did not significantly modify the observed results (Table 5).

**Figure 3:**
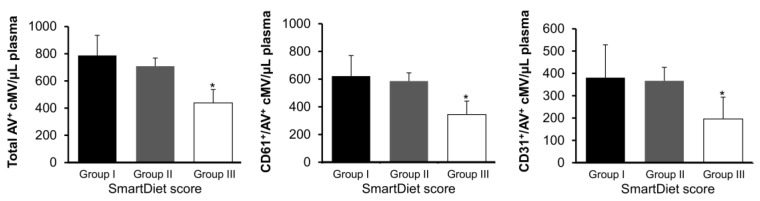
Concentration of circulating microvesicles according to groups of score from the SmartDiet questionnaire. Total cMV concentration (AV^+^), platelet-derived (CD61^+^/AV^+^) cMV, and cMV from activated cells (CD31^+^/AV^+^) concentrations in the three groups of scores. *Significantly different from the other groups (one-way ANOVA with the Bonferroni posthoc test). The markers used for cMV characterization are defined in Table 2. Group I was defined as ≤27points (*n* = 39, 22.4%), group II as 28–35 points (*n* = 106, 60.9%), and group III as ≥36 points (*n* = 29, 16.7%) in the questionnaire, where the higher the score, the healthier the diet, according to the Nordic Nutrition Recommendations. Here, cMV indicates circulating microvesicles, and AV is Annexin V.

As depicted in Figure 3, patients allocated at the highest score range (healthy diet) had significantly lower total (AV^+^) and platelet (CD61^+^/AV^+^ and CD31^+^/AV^+^) cMV (*p* = 0.028, 0.017, and 0.038, respectively, one-way ANOVA with the Bonferroni post hoc test) compared to patients allocated at the first and second tertiles of score.

## 4. Discussion

### 4.1. Previous Knowledge

A healthy diet should be the cornerstone of CVD prevention. In this setting, a high adherence to the Nordic diet has been related to reduced blood pressure [13], improved blood lipid profile [14] and insulin sensitivity [15], improved endothelial function [2], and reduced low-grade inflammation [16]. However, this is the first time that high adherence to the Nordic diet, assessed through the validated SmartDiet questionnaire (which was particularly developed to evaluate a subject’s diet regarding risk of CVD in the context of the Nordic diet), has been related to MV shedding.

### 4.2. Main Findings of the Study

The main finding in this cross-sectional study is that patients with higher adherence to the Nordic diet presented around 32% lower levels of total AV^+^ cMV, 41% lower levels of CD61^+^/AV^+^ cMV, and 30% lower levels of CD31^+^/AV^+^ cMV, compared to patients allocated to the first and second tertiles of score, despite being under dual antiplatelet therapy. No differences in cholesterol levels were observed across tertiles of score, probably because almost all patients were under statin treatment, as per guidelines after an AMI. Taking this into consideration, this inhibitory effect on platelet activation may contribute to the beneficial effects of the Nordic diet, which is associated with reduced mortality in patients with established CVD [17].

Interestingly, the mean score for each item was higher in group III compared to group II and group I, except for two food items, in which no differences were observed between groups of the SmartDiet score. This observation indicates that the group classification from the SmartDiet questionnaire, at least within the patients included in the study, reflected a dietary pattern rather than high/low consumption of a specific/single food group. Although the SmartDiet questionnaire has been validated by dietary fat and fiber [9], the protective effects of the Nordic diet may be considered to be a product of the sum of healthy consumption of single food items and should not be only attributed to a single food or food compound.

### 4.3. Review of Previous Data 

cMV were identified and quantified through AV binding, which has a high affinity for PS and therefore identifies PS externalization. AV^+^ cMV expose PS on their surfaces, providing a catalytic surface for factor X and prothrombin activation, thus promoting thrombin generation and clot formation [18]. Besides the externalization of PS, which confers procoagulant activity to cMV, cMV were also identified and quantified on the basis of antigens from their cell of origin and on markers of cell activation. In this line, platelet-derived cMV, which contain epitopes and biomolecules from platelets such as CD61 and/or CD31 and account for 74% of cMV AV^+^, amplify platelet activity on coagulation cascade and leukocyte (and platelet) adhesion/activation [19], promoting inflammation and increasing platelet deposition on damaged arteries and thrombus formation [18], thus increasing the atherothrombotic burden [7]. In fact, total AV^+^ and CD31^+^/AV^+^ cMV map and predict coronary atherosclerosis and calcification in familial hypercholesterolemia patients [20], contributing to an increased risk of a major adverse CV event. Therefore, nutritional strategies to decrease total AV^+^ and platelet-derived cMV release may improve CVD prognosis.

Few studies have analyzed the effects of diet on MV release. Cocoa flavonols have been shown to decrease CD31^+^ cMV concentrations in patients with coronary artery disease after one month of intake [21], as did one month of an intervention with the Mediterranean diet in elderly healthy individuals [22]. Supplementation with omega 3 fatty acids has been shown to reduce CD31^+^ [23] and CD61^+^ [24] cMV concentrations, and replacement of saturated fats with monounsaturated and omega 6 fatty acids reduced the number of CD31^+^ cMV [25]. Taking into consideration that the Nordic diet is rich in polyunsaturated fatty acids and polyphenols, our observations are endorsed by the literature.

### 4.4. Limitations and Scope of Future Studies

Our study is not exempt from limitations. The current study was performed in elderly patients with a relatively recent AMI, and therefore, these findings may not be extrapolated to healthy subjects or to other AMI populations. In addition, females accounted for only 29% of the study population, and thus sex-specific differences were not analyzed. This was an association study, and therefore it was hypothesis-generating. Although flow cytometry is the most widely used method for MV quantification and characterization, its limit of quantification is ranged between 0.1 and 0.3 µm of particle size. Therefore, MP populations below the limit of quantification were not considered and could be influenced by the Nordic diet quality. Considering all of these aspects, future studies should be focused on the evaluation of the effects of the Nordic diet on MV shedding from different sizes and cell origins through randomized trials, in order to confirm a cause–effect relationship and the potential sex-specific differences in the observed effects.

## 5. Conclusions

Dietary habits significantly affect MV shedding, known to directly influence cellular functions. This study showed that the higher the adherence to a healthy Nordic diet was, the lower the levels of total AV^+^ and platelet-derived AV^+^ cMV found in the circulating blood of Norwegian elderly patients with a recent AMI. The lower release of MV may potentially contribute to the beneficial effects of the Nordic diet.

## Figures and Tables

**Table 1:** Questions and scores of version 3 of the SmartDiet questionnaire used to assess dietary patterns and adherence to the Nordic diet.

	Least Healthy (1 point)	More Healthy (2 points)	Most Healthy (3 points)
Milk or yogurt			
What sort of milk do you use more often?	whole milk	low-fat milk or no milk at all	skimmed milk
Cream and sour cream			
What sort of cream and sour cream do you use more often?	whole cream	light cream	extra-light cream or less than once weekly consumption
Cheese			
What sort of cheese do you use more often?	white cheese	light cheese	cheese with rape and sunflower oil, extra-light cheese or less than once weekly consumption
Spreading/cured meat			
What sort of spreading meat do you use more often?	fat meats	-	lean meats or less than once weekly consumption
Meat for dinner			
What sort of meat do you consume for dinner more often?	Fat or processed meat	lean met with fat rim or skin	lean meats or less than once weekly consumption
Spreading fish			
How often do you spread fish in sandwiches or in salads for lunch?	up to 1 slice of bread per week or never	between 2 and 4 slices of bread per week	about 5 or more slices of bread per week
Fish for dinner			
How many times per week do you eat fish, fish products, and/or fish dishes?	between once per week and never	2 times per week	3 or more times per week
Mayonnaise, remoulade, and kaviar			
How often do you use mayonnaise products on bread food?	Up to 1 slice of bread per week or never or 8 or more times per week	2–7 slices of bread per week	-
Butter and margarine on bread food			
What sort of butter and margarine do you use more often?	dairy butter	margarine	light margarine or low consumption of butter and margarine
Fat in cooking			
What sort of fat do you use more often when frying, baking, or in sauce, such as dressings?	dairy butter	margarine	oils or use of fat for cooking
Bread and other grain products			
What sort of bread or grain cereals do you use more often?	white bread or low bread consumption	-	whole-grain bread and cereals
Vegetables, fruits and berries			
How many portions (150 g, equivalent to approximately 2 carrots or about a big apple) do you consume daily?	fewer than 2 portions (< 300 g)	2–4 portions (300–600 g)	more than 4 portions (> 600 g)
Sweet toppings and sweet drinks			
How often do you use sweet toppings or drinks with glucose or fructose?	3 or more times per day	2 times per day	0–1 times per day
Chocolate, snacks, cakes and biscuits			
How often do you eat snacks?	3 or more times per week	2 times per week	0–1 times per week

The points from each item are summed into a total SmartDiet score classified as < 27 points, unhealthy; 28–35 points, somewhat healthy; and >36 points, healthy diet.

**Table 2:** Cell molecules for circulating microvesicle (cMV) identification and characterization.

mAb	Alternative Name	Expression	Conjugation	Clone	Company
AV	PS-binding protein	Widely expressed	APC		BD Biosciences
CD142	Tissue Factor	Widely expressed	FITC	VD8	Sekisui diagnostics
*Platelet-Related*					
CD61	β_3_-integrin	Platelets	PE	VI-PL2	BD Pharmingen
CD31	PECAM-1	Activated Cells	FITC	AC128	Miltenyi Biotec
CD62P	P-Selectin	Platelets	PE	HI62P	Immunotools
CD42b	Glycoprotein Ib alpha chain	Platelets and megakaryocytes	PE	REA185	MACS Miltenyi
*Endothelial Cell-Related*					
CD146	Melanoma Cell Adhesion Molecule	Endothelial Cells	FITC	P1H12	BD Pharmingen
CD62E	E-Selectin	Endothelial Cells	PE	68-5H11	BD Biosciences
CD309	VEGFR-2	HSCs, EPCs, and ECs	FITC	ES8-20E6	MACS Miltenyi
*Erythrocyte-Related*					
CD235ab	Glycophorin A and B	Erythrocytes	FITC	HIR2	Immunotools
*Leukocyte-Related*					
CD45	Leukocyte Common Antigen	Leukocytes	PE	MEM-28	Immunotools
CD3	T-cell co-receptor	T-Lymphocytes	FITC	HIT3b	Immunotools
CD14	LPS-receptor	Macrophages, monocytes	PE	M5E2	BD Pharmingen
CD62L	L-Selectin	Leukocytes	FITC	LT-TD180	Immunotools
CD11b	Macrophage-1 Antigen (Mac-1)	Neutrophils, leukocytes	FITC	M1/70.15.11.5	MACS Miltenyi
*Smooth Muscle Cell-Related*					
SMA-α	Smooth Muscle Actin α	Smooth muscle cells	PE	1A4	R&D Systems

APC denotes allophycocyanin; AV, Annexin V; CD, cluster of differentiation; ECs, endothelial cells; EPCs, endothelial progenitor cells; FITC, fluorescein isothiocyanate; HSCs, hematopoietic stem cells; LPS, lipopolysaccharide; mAb indicates monoclonal antibody; PE, phycoerythrin; PECAM-1, platelet endothelial cell adhesion molecule; PS, phosphatidylserine; and VEGFR-2, vascular endothelial growth factor receptor-2.

**Table 3:** Characteristics of the total study population (*n* = 174) and according to the grouping by diet score.

Mean ± SD or *n* (%)	All Patients (*n* = 174)	Group I (*n* = 39)	Group II (*n =* 106)	Group III (*n* = 29)	*p*
Weeks after an AMI at inclusion					0.994
2 up to 4 weeks	90 (52)	21 (54)	53 (50)	16 (55)	
4 up to 6 weeks	41 (24)	9 (23)	25 (24)	7 (24)	
6–8 weeks	43 (25)	9 (23)	28 (26)	6 (21)	
Males	124 (71.2)	28 (71.8)	78 (73.6)	18 (62.1)	0.371
Age (years)	75 ± 4	75 ± 4	74 ± 4	73 ± 3	0.403
BMI (kg/m^2^)	26.11 ± 4.04	25.77 ± 3.68	26.33 ± 4.46	26.16 ± 3.54	0.803
Systolic blood pressure (mm Hg)	139 ± 9	138 ± 23	139 ± 18	130 ± 14	0.092
Diastolic blood pressure (mm Hg)	75 ± 10	74 ± 11	76 ± 8	74 ± 10	0.651
Total cholesterol (mmol/L)	3.88 ± 0.82	3.74 ± 0.90	3.87 ± 0.83	3.93 ± 0.74	0.410
HDL cholesterol (mmol/L)	1.38 ± 0.44	1.40 ± 0.52	1.37 ± 0.38	1.42 ± 0.45	0.973
Total cholesterol/HDL ratio	2.97 ± 0.85	2.79 ± 0.57	2.95 ± 0.82	2.95 ± 0.90	0.867
LDL cholesterol (mmol/L)	2.16 ± 0.64	2.05 ± 0.59	2.17 ± 0.64	2.2 ± 0.64	0.525
Triglycerides (mmol/L)	1.30 ± 0.84	1.20 ± 0.33	1.24 ± 0.57	1.40 ± 1.69	0.580
Glucose (mmol/L)	6.23 ± 1.97	5.93 ± 1.66	6.22 ± 1.89	5.76 ± 0.98	0.372
HbA1C (%)	6.14 ± 1.01	6.08 ± 0.78	6.19 ± 1.12	5.83 ± 0.71	0.246
Previous hypertension	103 (59.2)	26 (66.7)	56 (52.8)	21 (72.4)	0.400
Previous dyslipidemia	78 (44.8)	15 (38.5)	51 (48.1)	12 (41.4)	0.346
Previous diabetes	41 (23.6)	10 (25.6)	25 (23.6)	6 (20.7)	0.864
Current smokers	17 (9.8)	7 (17.9)	7 (6.6)	3 (10.3)	0.151
Medication after AMI					
Acetylsalicylic acid	166 (95.4)	35 (89.7)	102 (96.2)	29 (100)	0.228
ADP receptor inhibitors	163 (94.7)	36 (92.3)	100 (94.3)	27 (93.1)	0.885
Statins	170 (97.7)	38 (97.4)	103 (97.2)	29 (100)	0.692
Beta blockers	153 (87.9)	34 (87.1)	91 (85.8)	28 (96.5)	0.174
Calcium channel blockers	31 (17.8)	7 (17.9)	22 (20.7)	2 (6.9)	0.280
ACE inhibitors	58 (33.3)	13 (33.3)	36 (34.0)	9 (31.0)	0.931
ARB	44 (25.3)	8 (20.5)	30 (28.3)	6 (20.7)	0.522
Nitrates	21 (12.1)	3 (7.7)	16 (15.1)	2 (6.9)	0.283
Diuretics	41 (23.6)	8 (20.5)	29 (27.3)	4 (13.8)	0.132
Anticoagulants	23 (13.2)	7 (17.9)	12 (11.3)	4 (13.8)	0.503
Omega 3 supplements	88 (50.6)	20 (51.3)	52 (49.1)	16 (55.2)	0.456

*p* values refer to comparisons between groups of score from the ANOVA test for quantitative variables or the chi-squared test for categorical variables. Group I was defined as ≤ 27points, group II as 28–35 points, and group III as ≥ 36 points in the questionnaire, where the higher the score, the healthier the diet. ADP indicates adenosine diphosphate; ACE, angiotensin-converting enzyme; ARB, angiotensin II receptor blockers; AMI, acute myocardial infarction; BMI, body mass index; HbA1C, glycated hemoglobin; HDL, high-density lipoprotein; LDL, low-density lipoprotein.

**Table 4:** Mean of score for each food item according to groups of the SmartDiet score.

	*N* (%)			
	Group I (Unhealthy Diet)	Group II (Somewhat Unhealthy Diet)	Group III (Healthy Diet)	*p*
Milk or yogurt	2.0 ± 0.5 ^a^	2.2 ± 0.5 ^a,b^	2.7 ± 0.5 ^b^	0.001
Cream and sour cream	1.7 ± 0.8 ^a^	2.5 ± 0.6 ^b^	2.8 ± 0.4 ^b^	<0.0001
Cheese	1.1 ± 0.3 ^a^	1.6 ± 0.8 ^b^	2.7 ± 0.6 ^c^	<0.0001
Spreading/cured meat	1.7 ± 1.0 ^c^	2.6 ± 0.8 ^b^	3.0 ± 0.0 ^b^	<0.0001
Meat for dinner	1.7 ± 0.9 ^a^	2.6 ± 0.7 ^b^	3.0 ± 0.0 ^b^	<0.0001
Spreading fish	1.8 ± 0.7 ^a^	2.0 ± 0.7 ^a^	2.5 ± 0.7 ^b^	0.027
Fish for dinner	1.8 ± 0.7 ^a^	2.2 ± 0.7 ^b^	2.7 ± 0.4 ^c^	<0.0001
Mayonnaise, remoulade, and kaviar	1.4 ± 0.5	1.4 ± 0.5	1.4 ± 0.5	0.950
Butter and margarine on bread food	1.7 ± 1.0 ^a^	2.5 ± 0.8 ^b^	2.9 ± 0.3 ^b^	<0.0001
Fat in cooking	1.4 ± 0.8 ^a^	2.3 ± 0.9 ^b^	2.8 ± 0.4 ^c^	<0.0001
Bread and other grain products	1.5 ± 0.9 ^a^	2.5 ± 0.9 ^b^	2.7 ± 0.8 ^b^	<0.0001
Vegetables, fruits, and berries	1.5 ± 0.5 ^a^	1.8 ± 0.6 ^b^	2.2 ± 0.4 ^c^	0.003
Sweet toppings and sweet drinks	2.6 ± 0.5	2.7 ± 0.6	2.9 ± 0.3	0.259
Chocolate, snacks, cakes, and biscuits	2.1 ± 0.8 ^a^	2.5 ± 0.7 ^b^	3.0 ± 0.0 ^c^	0.001

The results are expressed as mean ± SD of the score (ranging from 1 to 3) for each food item within each group of diet quality classification. *p* is from the ANOVA with the Bonferroni post hoc test comparing the mean scores for each food item between groups of total SmartDiet score. Values in rows with different superscript letters are significantly different. Group I was defined as ≤ 27 points (*n* = 39, 22.4%), group II as 28–35 points (*n* = 106, 60.9%), and group III as ≥ 36 points (*n* = 29, 16.7%) as a product of the sum of the 14 items of the questionnaire, where the higher the score, the healthier the diet, according to the Nordic Nutrition Recommendations.

**Table 5:** Multivariable adjusted associations between the SmartDiet score and cMV.

SmartDiet Score				
cMV	*R^1^*	*p* ^1^	*β^2^*	*p^2^*
*Total cMV (AV^+^)*				
Unadjusted	−0.179	0.027	-	-
Multivariable adjusted*	0.275	0.031	−0.190	0.024
*Platelet-Derived cMV*				
CD61*^+^*/AV*^+^*				
Unadjusted	−0.198	0.014	-	-
Multivariable adjusted*	0.288	0.043	−0.229	0.006
CD31^+^/AV^+^				
Unadjusted	−0.168	0.037	-	-
Multivariable adjusted*	0.328	0.016	−0.179	0.033

^1^*R-* and *p*-values are from the ANOVA in the linear regression model, and *β^2^*- and *p*-values are for the contribution of the SmartDiet score in the multivariable adjusted model. *Adjusted for age, sex, use of omega 3 supplements, and smoking history. The markers used for microvesicle (MV) characterization are defined in Table 2. cMV indicates circulating microvesicles, and AV is Annexin V.
